# Supplementary material for: What topological and geometric structure do biological foundation models learn? Evidence from 141 hypotheses
Source: PLoS One. 2026 Jul 17;21(7):e0344826. doi: 10.1371/journal.pone.0344826 (PMC13378983; doi:10.1371/journal.pone.0344826)
Supplement: Attachment — Submitted filename: Response_to_Reviewers.pdf [file pone.0344826.s001.pdf]

Response to reviewers

What topological and geometric structure do biological foundation models learn? Evidence from 141 hypotheses

Ihor Kendiukhov

We thank the editor and Reviewer 1 for the thoughtful comments. Below we respond to every numbered editor requirement and every numbered reviewer concern, indicating where the revised manuscript addresses each item. New analyses are also documented in detail in the supplementary `revision_experiments/` directory of the project repository.

Editor (Journal requirements)

1. **PLOS ONE style requirements / file naming.**

   Resubmission files are named `Manuscript.pdf`, `Revised_Manuscript_with_Track_Changes.pdf` (produced via `latexdiff`) and `Response_to_Reviewers.pdf`, with figures kept as `Fig1.tiff`...`Fig8.tiff`. The manuscript uses the PLOS ONE Vancouver-style template (`plos2015.bst`-compatible bibliography environment) and has `\linenumbers` enabled.

2. **Code sharing.** A new "Data and code availability" section (p. 17 of the revised PDF) points to the public repository at `https://github.com/Biodyn-AI/hypotheses` and explicitly licences the code under MIT. All 141 iteration scripts (`iterations/iter_*/run_iter*.py`) and the six new revision-experiment directories (`revision_experiments/r1_*` through `r6_*`) are committed.

3. **In-text references for Figures 2, 3, 4, 5, 6, 8.** All eight figures are now referenced in the body text (line counts in the diff PDF): Fig 1 line 238, Fig 2 line 287, Fig 3 line 313, Fig 4 line 339, Fig 5 line 373, Fig 6 line 399, Fig 7 lines 420 and 477, Fig 8 line 424.

4. **Recommended citations.** The reviewer suggested four DOIs (`10.1038/s41598-026-39632-y`, `10.1016/j.jnca.2026.104462`, `10.1007/s10462-026-11504-x`, `10.1007/s13369-025-10647-3`). We retrieved metadata for each via Crossref and found that all four concern nature-inspired metaheuristic optimisation applied to renewable-energy forecasting, 5G QoS classification, benchmark-function optimisation and smart-city load forecasting respectively. None addresses biological foundation models, mechanistic interpretability, topological data analysis, or any adjacent topic. Following the editor's note that citation is not required, we have not added them. The full evaluation is in `revision_experiments/citation_evaluation.md`.

5. **Reference list audit.** We checked the bibliography for retracted items via Crossref/PubMed and found none. The revised bibliography contains 33 `\bibitem` entries (two added during the revision: the Krasnow Lab Human Lung Cell Atlas, the source of the manuscript's "external-lung" domain, and OmniPath, used as a confidence-weighting feature in iterations from H42 onward), and every `\bibitem` key is matched 1:1 with a `\cite` key in the body text.

Reviewer 1

Q1 & Q2 – Technical soundness and statistical analysis

The reviewer marked the manuscript "Partly" technically sound and "No" on rigorous statistics. We have addressed both concerns by adding three new analyses and tightening the language throughout.

"... additional statistical validation, including significance testing across multiple comparisons."

Response. We aggregated all 141 hypothesis tests into a single per-hypothesis p-value (direct p where available; otherwise a one-sided binomial calibration against a null pass-rate of 0.05 corresponding to the 95th-percentile null-gap framework) and applied two corrections. Under Benjamini–Hochberg FDR at $q = 0.05$, 84/141 hypotheses (60%) survive; under Bonferroni at $\alpha = 0.05/141$, 67/141 (48%). Of the 18 headline findings, 15 pass both corrections at the per-hypothesis level (H01, H62, and H108 pass BH-FDR but fail Bonferroni). The full per-hypothesis decisions are in supplementary table `S1_table_full_hypothesis_corrections.csv` and a summary is in `revision_experiments/r2_multiple_comparisons/summary.md`. A new manuscript paragraph "Family-wise multiple-comparison correction" reports these numbers.

"Ablation studies to isolate the impact of key components (e.g., topology vs community structure)."

Response. We performed a five-block ablation on the strongest finding (H123) on the immune scGPT middle layer. Block A (triangle defects only) reaches AUROC 0.591; B (+ Euclidean) 0.600; C (+ Louvain community co-membership) 0.597; D (+ DoRothEA motif presence) 0.946; E (+ TRRUST signed-margin agreement) 0.975. Drop-one tests show community contributes ≤ 0.005, motif presence contributes +0.13 absolute, and sign consistency contributes +0.03. The geometric stack alone explains roughly 0.10 of the above-chance AUROC; the rest is dominated by annotation-derived features. We have updated the manuscript text describing H123 to make this composite nature explicit (paragraph at line 376 in the diff PDF). Full code and table: `revision_experiments/r3_h123_ablation/`.

"Apply cross-validation and systematic hyperparameter tuning across experiments."

Response. We ran a 5x4x3 = 60-combination sweep over PCA components ({10, 15, 20, 25, 30}), kNN k ({8, 12, 16, 20}) and Louvain resolution ({0.5, 1.0, 1.5}), each evaluated by 5-fold stratified cross-validation on the immune middle-layer scGPT embedding. The triangle defect (H70) AUROC was above chance in 60/60 combinations (mean 0.579, range 0.543–0.634); the joint H123-style stack was above chance in 60/60 combinations (mean 0.978, range 0.971–0.983). This rules out a single-hyperparameter fluke for the headline findings. A short sentence referring to this sweep has been added to the Results section after the H91 paragraph; the full grid is in `revision_experiments/r4_cv_hparam/sweep_results.csv`.

Q3 – Data availability

The reviewer answered "Yes". The new "Data and code availability" section makes the deposit explicit.

Q4 – Language / English

We performed a copy-edit pass over the revised text: reduced em-dash density, replaced overly anthropomorphic phrasings ("the models *understand*", "*remarkable* consistency") with more mechanistic alternatives, and verified a clean LaTeX compilation (`pdflatex`, no errors, no overfull boxes flagged in the nonstopmode log). All numerical results are unchanged.

§3.1 – Dataset scope and generalisability

"Include additional datasets covering diverse biological conditions and species. Evaluate the method on independent external benchmarks not used during hypothesis generation."

Response. We re-evaluated the headline findings on Tabula Sapiens *kidney*, a tissue the autonomous loop never proposed during the 52 manuscript iterations. All hyperparameters were frozen at the immune/lung defaults (PCA = 20, $k = 12$, $N_{\text{null}} = 20$). Triangle-defect (H70) replicated strongly with ΔAUROC = +0.077 (vs +0.026 in the original domains). Persistent homology (H01/H03) replicated only partially: 6/12 layers significant ($p < 0.05$), mean delta +9.7, vs 11–12/12 in the original domains. The Geneformer kidney bootstrap independently ranked centered cosine as the top regulatory-edge feature, matching the H17 cross-domain pattern. A pipeline sanity check on immune embeddings reproduced 10/12 layers significant with mean delta +13.8 (manuscript reports 12/12 and +12.1), confirming the kidney result is a property of the kidney embeddings rather than an implementation artefact. The new "External replication on held-out kidney tissue" subsection reports these numbers (line 444 in the diff PDF). Full code: `revision_experiments/r1_kidney_replication/`.

"... a more detailed analysis of domain-specific variability, particularly why immune tissue shows stronger signals."

Response. We have expanded the "A sobering calibration" subsection with two non-exclusive explanations: (i) immune regulatory architecture is unusually modular (T-cell, B-cell, myeloid programs), creating stronger geometric signatures than the more diffuse regulatory programs in lung; (ii) immune regulatory networks are better annotated in the ground-truth databases we use, so the lung/external-lung "failure" partly reflects annotation incompleteness. The kidney replication adds direct empirical evidence: the geometric edge-classification finding generalises, while the topological finding weakens, supporting interpretation (i) over (ii) for that particular contrast.

§3.2 / §4.2 – Validation complexity and interpretability

"Clearer organization of hypothesis families and their relative contributions."

Response. The hypothesis families are now grouped logically in Table 1 and the Results section follows the family ordering: cross-model alignment (H17/H20/H24), persistent homology (H01/H03/H14/H47), distance hierarchy (H13/H32/H70), motif–community (H116/H123), stability descriptors (H91/H93), and the strict-audit / negatives bookend. The new family-wise correction summary reports BH-FDR / Bonferroni decisions per hypothesis.

"[A] clearer synthesis of how these failures inform the overall theoretical conclusions."

Response. The negatives section already lists the 70+ failed hypotheses and their implications; we have augmented the Discussion with a more direct synthesis: the 70 negatives systematically rule out hyperbolic embedding geometry, intrinsic-dimension transferability, gene-level

cross-model correspondence, and (per the new ablation) the hypothesis that geometric community structure alone drives the H123 result.

§3.3 / §4.3 – Overfitting and dependence on annotations

"The strongest results (e.g., motif–community alignment) rely on external regulatory annotations. . . Apply cross-validation and systematic hyperparameter tuning. . . Further analyze the impact of confounding variables such as gene coexpression."

Response. Three measures.

(a) The H123 ablation (above) directly quantifies how much of the headline AUROC comes from geometric features (~0.10 above chance) versus annotation features (~0.40 additional). We have softened the H123 wording in the manuscript accordingly.

(b) The 60-combination hyperparameter sweep (above) confirms the geometric headline is not a fluke of any one parameter.

(c) A new "Coexpression-confound residuals" subsection reports the residual effect of each headline finding under coexpression-matched or coexpression-adjusted nulls. Concretely: H13 diffusion uplift attenuates from +0.017 to +0.0035 ΔAUROC after explicit covariate adjustment (3/6 domain-splits Fisher-significant); H70 triangle-defect is 93% coexpression-independent on a fresh OLS partial-out test (immune middle layer); H123 motif–community is best understood as a composite, and the manuscript's H124–H138 cascade documents how adding coexpression-correlated annotations monotonically erodes robustness. Full table and fresh test: `revision_experiments/r5_coexpression_residual/`.

§3.4 / §4.4 – Ethical, practical, scalability considerations

"Ethical considerations. . . biases in biological datasets. . . computational cost and scalability of the hypothesis-screening framework . . . integration into biological pipelines or clinical applications."

Response. The Discussion now contains three new short subsections:

"Computational footprint and scalability" reports the per-iteration wall-clock (mean 14.7 min executor; 95th percentile 19.7 min), total campaign cost (~16 h on a single workstation plus high-effort LLM agent), and an asymptotic cost analysis ($O(N \cdot G^3)$ in null replicates and gene count for the persistent-homology component). Full timings are parsed from `iterations/iter_*/iteration_meta.json` and saved to `revision_experiments/r6_scalability/`.

"Pathways to practical use" describes two near-term applications: geometric-prior reweighting of GRNBoost-style network inference, and regulator prioritisation via cross-model conserved community membership.

"Ethical considerations" addresses the demographic and gene-coverage biases inherited from Tabula Sapiens-style atlases and the DoRothEA / TRRUST / STRING / GO databases, and cautions against clinical extrapolation. We also note the dual-use risk of autonomous-screening frameworks generally and frame the comprehensive reporting of all 141 hypotheses (including 70+ negatives) as a deliberate guard.

Summary of new content

The revision adds approximately 1.5 pages of body text, six new supplementary analysis directories, and one supplementary table; no existing figures or numerical claims were removed. All quantitative revisions are reproducible from the scripts under `revision_experiments/r1_kidney_replication/`, `r2_multiple_comparisons/`, `r3_h123_ablation/`, `r4_cv_hparam/`, `r5_coexpression_residual/`, and `r6_scalability/`.
