# Supplementary material for: What topological and geometric structure do biological foundation models learn? Evidence from 141 hypotheses
Source: PLoS One. 2026 Jul 17;21(7):e0344826. doi: 10.1371/journal.pone.0344826 (PMC13378983; doi:10.1371/journal.pone.0344826)
Supplement: Peer review history

# Peer review: Decision Letter 0

**DOI:** 10.1371/journal.pone.0344826.r001

**Authors:** Alexander Kolpakov

5 May 2026

PONE-D-26-09692

What topological and geometric structure do biological foundation models learn? Evidence from 141 hypotheses

PLOS One

Dear Dr. Kendiukhov,

Thank you for submitting your manuscript to PLOS ONE. After careful consideration, we feel that it has merit but does not fully meet PLOS ONE’s publication criteria as it currently stands. Therefore, we invite you to submit a revised version of the manuscript that addresses the points raised during the review process.

Please submit your revised manuscript by Jun 19 2026 11:59PM. If you will need more time than this to complete your revisions, please reply to this message or contact the journal office at plosone@plos.org. When you're ready to submit your revision, log on to https://www.editorialmanager.com/pone/ and select the 'Submissions Needing Revision' folder to locate your manuscript file.

Please include the following items when submitting your revised manuscript:

- A letter that responds to each point raised by the academic editor and reviewer(s). You should upload this letter as a separate file labeled 'Response to Reviewers'.
- A marked-up copy of your manuscript that highlights changes made to the original version. You should upload this as a separate file labeled 'Revised Manuscript with Track Changes'.
- An unmarked version of your revised paper without tracked changes. You should upload this as a separate file labeled 'Manuscript'.

If you would like to make changes to your financial disclosure, please include your updated statement in your cover letter. Guidelines for resubmitting your figure files are available below the reviewer comments at the end of this letter.

If applicable, we recommend that you deposit your laboratory protocols in protocols.io to enhance the reproducibility of your results. Protocols.io assigns your protocol its own identifier (DOI) so that it can be cited independently in the future. For instructions see: https://journals.plos.org/plosone/s/submission-guidelines#loc-laboratory-protocols. Additionally, PLOS ONE offers an option for publishing peer-reviewed Lab Protocol articles, which describe protocols hosted on protocols.io. Read more information on sharing protocols at https://plos.org/protocols?utm_medium=editorial-email&utm_source=authorletters&utm_campaign=protocols.

As the corresponding author, your ORCID iD is verified in the submission system and will appear in the published article. PLOS supports the use of ORCID, and we encourage all coauthors to register for an ORCID iD and use it as well. Please encourage your coauthors to verify their ORCID iD within the submission system before final acceptance, as unverified ORCID iDs will not appear in the published article. *Only*  the individual author can complete the verification step; PLOS staff *cannot*  verify ORCID iDs on behalf of authors.

We look forward to receiving your revised manuscript.

Kind regards,

Alexander Kolpakov

Academic Editor

PLOS One

Journal Requirements:

When submitting your revision, we need you to address these additional requirements.

1. Please ensure that your manuscript meets PLOS ONE's style requirements, including those for file naming. The PLOS ONE style templates can be found at

https://journals.plos.org/plosone/s/file?id=wjVg/PLOSOne_formatting_sample_main_body.pdf and https://journals.plos.org/plosone/s/file?id=ba62/PLOSOne_formatting_sample_title_authors_affiliations.pdf

2. Please note that PLOS One has specific guidelines on code sharing for submissions in which author-generated code underpins the findings in the manuscript. In these cases, we expect all author-generated code to be made available without restrictions upon publication of the work. Please review our guidelines at https://journals.plos.org/plosone/s/materials-and-software-sharing#loc-sharing-code and ensure that your code is shared in a way that follows best practice and facilitates reproducibility and reuse.

3. Please ensure that you refer to Figure 2, 3, 4, 5, 6, 8 in your text as, if accepted, production will need this reference to link the reader to the figure.

4. If the reviewer comments include a recommendation to cite specific previously published works, please review and evaluate these publications to determine whether they are relevant and should be cited. There is no requirement to cite these works unless the editor has indicated otherwise.

5. Please review your reference list to ensure that it is complete and correct. If you have cited papers that have been retracted, please include the rationale for doing so in the manuscript text, or remove these references and replace them with relevant current references. Any changes to the reference list should be mentioned in the rebuttal letter that accompanies your revised manuscript. If you need to cite a retracted article, indicate the article’s retracted status in the References list and also include a citation and full reference for the retraction notice.

Additional Editor Comments :

It can be accepted after the minor revision recommended by the referee.

[Note: HTML markup is below. Please do not edit.]

Reviewers' comments:

Reviewer's Responses to Questions

**Comments to the Author**

1. Is the manuscript technically sound, and do the data support the conclusions?

The manuscript must describe a technically sound piece of scientific research with data that supports the conclusions. Experiments must have been conducted rigorously, with appropriate controls, replication, and sample sizes. The conclusions must be drawn appropriately based on the data presented.

Reviewer #1: Partly

**********

2. Has the statistical analysis been performed appropriately and rigorously?

Reviewer #1: No

**********

3. Have the authors made all data underlying the findings in their manuscript fully available?

The PLOS Data policy requires authors to make all data underlying the findings described in their manuscript fully available without restriction, with rare exception (please refer to the Data Availability Statement in the manuscript PDF file). The data should be provided as part of the manuscript or its supporting information, or deposited to a public repository. For example, in addition to summary statistics, the data points behind means, medians and variance measures should be available. If there are restrictions on publicly sharing data—e.g. participant privacy or use of data from a third party—those must be specified.

Reviewer #1: Yes

**********

4. Is the manuscript presented in an intelligible fashion and written in standard English?

PLOS ONE does not copyedit accepted manuscripts, so the language in submitted articles must be clear, correct, and unambiguous. Any typographical or grammatical errors should be corrected at revision, so please note any specific errors here.

Reviewer #1: No

**********

5. Review Comments to the Author

Please use the space provided to explain your answers to the questions above. You may also include additional comments for the author, including concerns about dual publication, research ethics, or publication ethics. (Please upload your review as an attachment if it exceeds 20,000 characters)

Reviewer #1: 1. Summary of the Manuscript

The manuscript entitled “What topological and geometric structure do biological foundation models learn? Evidence from 141 hypotheses” investigates whether biological foundation models such as scGPT and Geneformer encode meaningful geometric and topological structures within their internal representations. The study employs an autonomous hypothesis-generation and testing framework to evaluate 141 hypotheses across multiple experimental conditions, datasets, and null models. The authors aim to distinguish genuine biological structure from statistical artifacts through rigorous validation and extensive null-model comparisons.

The work lies at the intersection of mechanistic interpretability, computational biology, and topological data analysis, addressing an important question regarding the interpretability and reliability of modern biological foundation models.

2. Strong Aspects

2.1 Innovativeness and Conceptual Contribution

The manuscript demonstrates a high degree of innovation, particularly through its use of an autonomous executor–brainstormer loop to systematically generate and evaluate hypotheses. This approach represents a significant methodological advancement compared to traditional hypothesis-driven research, as it allows exploration of a vast hypothesis space while explicitly documenting both positive and negative results.

Furthermore, the integration of advanced analytical techniques—such as persistent homology, manifold distance metrics, canonical correlation analysis, and community detection—reflects a sophisticated and interdisciplinary approach. The combination of topological data analysis with biological foundation models is especially noteworthy, as it extends interpretability research beyond linear geometric representations into nonlinear and higher-order structures.

2.2 Methodological Rigor and Data Analysis

The study is methodologically robust and carefully structured. Key strengths include:

The use of multiple datasets and tissue domains (immune, lung, and external-lung), which supports cross-domain validation.

Implementation of disjoint gene-pool splits to prevent information leakage, enhancing the reliability of generalization claims.

A hierarchical null-model framework, ranging from feature shuffling to strict max-null auditing, which provides a rigorous benchmark for distinguishing real signal from artifacts.

The inclusion of multiple evaluation metrics such as AUROC improvements, null-gap analysis, and domain-split pass rates further strengthens the analytical depth of the study.

2.3 Performance and Empirical Findings

The results demonstrate that the proposed analytical framework successfully identifies meaningful geometric and topological structures:

Strong cross-model alignment (canonical correlation ≈ 0.80) indicates that independently trained models converge on similar geometric representations.

Persistent homology analysis reveals statistically significant topological features across most transformer layers, suggesting non-trivial structural organization in embedding spaces.

The identification of a hierarchy of distance metrics, where manifold-based distances outperform Euclidean distance, provides valuable insight into how biological relationships are encoded.

Additionally, the discovery that regulatory motifs align with geometric community structure represents a particularly strong and biologically meaningful finding.

2.4 Practical Relevance and Impact

The study has substantial implications for both computational biology and machine learning:

It advances understanding of how foundation models encode biological knowledge, which is critical for trust and interpretability.

The findings suggest potential applications in gene regulatory network inference, feature selection, and biological discovery.

The systematic mapping of both positive and negative results contributes to reducing publication bias and improving reproducibility in interpretability research.

Overall, the work bridges theoretical modeling and practical biological insight, making it highly relevant for real-world applications.

3. Weak Aspects

3.1 Dataset Scope and Generalizability

Despite the use of multiple tissue domains, the study relies on a limited number of datasets and focuses primarily on specific biological contexts. The results show that strong signals are concentrated in immune tissue, while lung and external-lung domains exhibit weaker or fragile effects under strict validation.

This raises concerns about the generalizability of the findings across broader biological systems. The observed domain dependency suggests that conclusions may not extend uniformly to other tissues or datasets.

3.2 Validation Complexity and Interpretability

While the hierarchical null-model framework is a strength, it also introduces complexity that may hinder interpretability. The large number of hypotheses (141) and multiple layers of validation make it challenging to clearly isolate the contribution of individual methodological components.

Moreover, although negative results are documented, the manuscript could provide a clearer synthesis of how these failures inform the overall theoretical conclusions.

3.3 Overfitting and Dependence on External Annotations

There is evidence that some performance improvements may be partially driven by confounding factors:

The reduction in performance under coexpression-matched null models suggests that some geometric signals may reflect known biological correlations rather than novel insights.

The strongest results (e.g., motif–community alignment) rely on external regulatory annotations, indicating that the model does not independently encode all relevant biological information.

Additionally, the degradation of robustness when incorporating additional biological annotations highlights potential overfitting or correlation with null structures.

3.4 Missing Ethical, Practical, and Scalability Considerations

The manuscript does not sufficiently address broader implications:

Ethical considerations, such as biases in biological datasets or downstream decision-making risks, are not discussed.

Computational cost and scalability of the hypothesis-screening framework are not evaluated, despite its iterative and resource-intensive nature.

Practical deployment challenges, including integration into biological pipelines or clinical applications, are not explored.

These omissions limit the applicability of the study in real-world contexts.

4. Recommended Changes

4.1 Expand Dataset Diversity and Validation Scope

To improve generalizability, the authors should:

Include additional datasets covering diverse biological conditions and species.

Evaluate the method on independent external benchmarks not used during hypothesis generation.

Provide a more detailed analysis of domain-specific variability, particularly why immune tissue shows stronger signals.

4.2 Strengthen Validation and Interpretability

The manuscript would benefit from:

Clearer organization of hypothesis families and their relative contributions.

Additional statistical validation, including significance testing across multiple comparisons.

Ablation studies to isolate the impact of key components (e.g., topology vs. community structure).

4.3 Improve Robustness and Reduce Overfitting Risks

To enhance methodological robustness, the authors are encouraged to:

Apply cross-validation and systematic hyperparameter tuning across experiments.

Incorporate regularization strategies or noise-based validation to test stability.

Further analyze the impact of confounding variables such as gene coexpression.

4.4 Address Ethical and Practical Considerations

The manuscript should include:

A discussion on ethical implications, including potential biases in biological data and interpretability risks.

Evaluation of computational complexity and scalability of the proposed framework.

Consideration of how the approach can be integrated into practical biological or biomedical workflows.

suggested citation: https://doi.org/10.1038/s41598-026-39632-y https://doi.org/10.1016/j.jnca.2026.104462 https://doi.org/10.1007/s10462-026-11504-x https://doi.org/10.1007/s13369-025-10647-3

**********

6. PLOS authors have the option to publish the peer review history of their article (what does this mean?). If published, this will include your full peer review and any attached files.

If you choose “no”, your identity will remain anonymous but your review may still be made public.

**Do you want your identity to be public for this peer review?**  For information about this choice, including consent withdrawal, please see our Privacy Policy.

Reviewer #1: No

**********

[NOTE: If reviewer comments were submitted as an attachment file, they will be attached to this email and accessible via the submission site. Please log into your account, locate the manuscript record, and check for the action link "View Attachments". If this link does not appear, there are no attachment files.]

To ensure your figures meet our technical requirements, please review our figure guidelines: https://journals.plos.org/plosone/s/figures

You may also use PLOS’s free figure tool, NAAS, to help you prepare publication quality figures: https://journals.plos.org/plosone/s/figures#loc-tools-for-figure-preparation.

NAAS will assess whether your figures meet our technical requirements by comparing each figure against our figure specifications.

# Author comment: Author response to Decision Letter 1

**DOI:** 10.1371/journal.pone.0344826.r002

10 Jun 2026

Manuscript: "What topological and geometric structure do biological foundation

models learn? Evidence from 141 hypotheses"

Author: Ihor Kendiukhov

Journal: PLOS ONE

We thank the editor and Reviewer 1 for the thoughtful comments. Below we

respond to every numbered editor requirement and every numbered reviewer

concern, indicating where the revised manuscript addresses each item. New

analyses are also documented in detail in the supplementary

revision_experiments/ directory of the project repository at

https://github.com/Biodyn-AI/hypotheses.

================================================================================

EDITOR (Journal requirements)

================================================================================

1. PLOS ONE style requirements / file naming.

Resubmission files are named Manuscript.pdf,

Revised_Manuscript_with_Track_Changes.pdf (produced via latexdiff) and

Response_to_Reviewers.pdf, with figures kept as Fig1.tiff ... Fig8.tiff.

The manuscript uses the PLOS ONE Vancouver-style template

(plos2015.bst-compatible bibliography environment) and has \linenumbers

enabled.

2. Code sharing.

A new "Data and code availability" section (p. 17 of the revised PDF)

points to the public repository at https://github.com/Biodyn-AI/hypotheses

and explicitly licences the code under MIT. All 141 iteration scripts

(iterations/iter_*/run_iter*.py) and the six new revision-experiment

directories (revision_experiments/r1_* through r6_*) are committed.

3. In-text references for Figures 2, 3, 4, 5, 6, 8.

All eight figures are now referenced in the body text (line counts in

the diff PDF):

Fig 1 line 238

Fig 2 line 287

Fig 3 line 313

Fig 4 line 339

Fig 5 line 373

Fig 6 line 399

Fig 7 lines 420 and 477

Fig 8 line 424

4. Recommended citations.

The reviewer suggested four DOIs:

10.1038/s41598-026-39632-y

10.1016/j.jnca.2026.104462

10.1007/s10462-026-11504-x

10.1007/s13369-025-10647-3

We retrieved metadata for each via Crossref and found that all four

concern nature-inspired metaheuristic optimisation applied to renewable-

energy forecasting, 5G QoS classification, benchmark-function optimisation

and smart-city load forecasting respectively. None addresses biological

foundation models, mechanistic interpretability, topological data

analysis, or any adjacent topic. Following the editor's note that

citation is not required, we have not added them. The full evaluation is

in revision_experiments/citation_evaluation.md.

5. Reference list audit.

We checked the bibliography for retracted items via Crossref/PubMed and

found none. The revised bibliography contains 33 \bibitem entries (two

added during the revision: the Krasnow Lab Human Lung Cell Atlas, the

source of the manuscript's "external-lung" domain, and OmniPath, used

as a confidence-weighting feature in iterations from H42 onward), and

every \bibitem key is matched 1:1 with a \cite key in the body text.

================================================================================

REVIEWER 1

================================================================================

--------------------------------------------------------------------------------

Q1 & Q2 -- Technical soundness and statistical analysis

--------------------------------------------------------------------------------

The reviewer marked the manuscript "Partly" technically sound and "No" on

rigorous statistics. We have addressed both concerns by adding three new

analyses and tightening the language throughout.

REVIEWER COMMENT

"...additional statistical validation, including significance testing

across multiple comparisons."

RESPONSE

We aggregated all 141 hypothesis tests into a single per-hypothesis

p-value (direct p where available; otherwise a one-sided binomial

calibration against a null pass-rate of 0.05 corresponding to the

95th-percentile null-gap framework) and applied two corrections. Under

Benjamini-Hochberg FDR at q = 0.05, 84/141 hypotheses (60%) survive;

under Bonferroni at alpha = 0.05/141, 67/141 (48%). Of the 18 headline

findings, 15 pass both corrections at the per-hypothesis level (H01,

H62, and H108 pass BH-FDR but fail Bonferroni). The full per-hypothesis

decisions are in supplementary table

S1_table_full_hypothesis_corrections.csv and a summary is in

revision_experiments/r2_multiple_comparisons/summary.md. A new

manuscript paragraph "Family-wise multiple-comparison correction"

reports these numbers.

REVIEWER COMMENT

"Ablation studies to isolate the impact of key components (e.g.,

topology vs community structure)."

RESPONSE

We performed a five-block ablation on the strongest finding (H123) on

the immune scGPT middle layer. Block A (triangle defects only) reaches

AUROC 0.591; B (+ Euclidean) 0.600; C (+ Louvain community

co-membership) 0.597; D (+ DoRothEA motif presence) 0.946; E (+ TRRUST

signed-margin agreement) 0.975. Drop-one tests show community

contributes <= 0.005, motif presence contributes +0.13 absolute, and

sign consistency contributes +0.03. The geometric stack alone explains

roughly 0.10 of the above-chance AUROC; the rest is dominated by

annotation-derived features. We have updated the manuscript text

describing H123 to make this composite nature explicit (paragraph at

line 376 in the diff PDF). Full code and table:

revision_experiments/r3_h123_ablation/.

REVIEWER COMMENT

"Apply cross-validation and systematic hyperparameter tuning across

experiments."

RESPONSE

We ran a 5x4x3 = 60-combination sweep over PCA components

({10, 15, 20, 25, 30}), kNN k ({8, 12, 16, 20}) and Louvain resolution

({0.5, 1.0, 1.5}), each evaluated by 5-fold stratified cross-validation

on the immune middle-layer scGPT embedding. The triangle defect (H70)

AUROC was above chance in 60/60 combinations (mean 0.579, range

0.543-0.634); the joint H123-style stack was above chance in 60/60

combinations (mean 0.978, range 0.971-0.983). This rules out a

single-hyperparameter fluke for the headline findings. A short sentence

referring to this sweep has been added to the Results section after the

H91 paragraph; the full grid is in

revision_experiments/r4_cv_hparam/sweep_results.csv.

--------------------------------------------------------------------------------

Q3 -- Data availability

--------------------------------------------------------------------------------

The reviewer answered "Yes". The new "Data and code availability" section

makes the deposit explicit.

--------------------------------------------------------------------------------

Q4 -- Language / English

--------------------------------------------------------------------------------

We performed a copy-edit pass over the revised text: reduced em-dash

density, replaced overly anthropomorphic phrasings ("the models

*understand*", "*remarkable* consistency") with more mechanistic

alternatives, and verified a clean LaTeX compilation (pdflatex, no errors,

no overfull boxes flagged in the nonstopmode log). All numerical results

are unchanged.

--------------------------------------------------------------------------------

Section 3.1 -- Dataset scope and generalisability

--------------------------------------------------------------------------------

REVIEWER COMMENT

"Include additional datasets covering diverse biological conditions

and species. Evaluate the method on independent external benchmarks

not used during hypothesis generation."

RESPONSE

We re-evaluated the headline findings on Tabula Sapiens *kidney*, a

tissue the autonomous loop never proposed during the 52 manuscript

iterations. All hyperparameters were frozen at the immune/lung defaults

(PCA = 20, k = 12, N_null = 20). Triangle-defect (H70) replicated

strongly with delta-AUROC = +0.077 (vs +0.026 in the original domains).

Persistent homology (H01/H03) replicated only partially: 6/12 layers

significant (p < 0.05), mean delta +9.7, vs 11-12/12 in the original

domains. The Geneformer kidney bootstrap independently ranked centered

cosine as the top regulatory-edge feature, matching the H17 cross-

domain pattern (a full scGPT-vs-Geneformer feature-level concordance

test on kidney was descoped from this revision). A pipeline sanity

check on immune embeddings reproduced 10/12 layers significant with

mean delta +13.8 (manuscript reports 12/12 and +12.1), confirming the

kidney result is a property of the kidney embeddings rather than an

implementation artefact. The new "External replication on held-out

kidney tissue" subsection reports these numbers (line 444 in the diff

PDF). Full code: revision_experiments/r1_kidney_replication/.

REVIEWER COMMENT

"...a more detailed analysis of domain-specific variability,

particularly why immune tissue shows stronger signals."

RESPONSE

We have expanded the "A sobering calibration" subsection with two

non-exclusive explanations: (i) immune regulatory architecture is

unusually modular (T-cell, B-cell, myeloid programs), creating stronger

geometric signatures than the more diffuse regulatory programs in lung;

(ii) immune regulatory networks are better annotated in the ground-

truth databases we use, so the lung/external-lung "failure" partly

reflects annotation incompleteness. The kidney replication adds direct

empirical evidence: the geometric edge-classification finding

generalises, while the topological finding weakens, supporting

interpretation (i) over (ii) for that particular contrast.

--------------------------------------------------------------------------------

Section 3.2 / 4.2 -- Validation complexity and interpretability

--------------------------------------------------------------------------------

REVIEWER COMMENT

"Clearer organization of hypothesis families and their relative

contributions."

RESPONSE

The hypothesis families are now grouped logically in Table 1 and the

Results section follows the family ordering: cross-model alignment

(H17/H20/H24), persistent homology (H01/H03/H14/H47), distance

hierarchy (H13/H32/H70), motif-community (H116/H123), stability

descriptors (H91/H93), and the strict-audit / negatives bookend. The

new family-wise correction summary reports BH-FDR / Bonferroni

decisions per hypothesis.

REVIEWER COMMENT

"[A] clearer synthesis of how these failures inform the overall

theoretical conclusions."

RESPONSE

The negatives section already lists the 70+ failed hypotheses and

their implications; we have augmented the Discussion with a more

direct synthesis: the 70 negatives systematically rule out hyperbolic

embedding geometry, intrinsic-dimension transferability, gene-level

cross-model correspondence, and (per the new ablation) the hypothesis

that geometric community structure alone drives the H123 result.

--------------------------------------------------------------------------------

Section 3.3 / 4.3 -- Overfitting and dependence on annotations

--------------------------------------------------------------------------------

REVIEWER COMMENT

"The strongest results (e.g., motif-community alignment) rely on

external regulatory annotations... Apply cross-validation and

systematic hyperparameter tuning... Further analyze the impact of

confounding variables such as gene coexpression."

RESPONSE

Three measures.

(a) The H123 ablation (above) directly quantifies how much of the

headline AUROC comes from geometric features (~0.10 above chance)

versus annotation features (~+0.40 additional). We have softened

the H123 wording in the manuscript accordingly.

(b) The 60-combination hyperparameter sweep (above) confirms the

geometric headline is not a fluke of any one parameter.

(c) A new "Coexpression-confound residuals" subsection reports the

residual effect of each headline finding under coexpression-

matched or coexpression-adjusted nulls. Concretely: H13 diffusion

uplift attenuates from +0.017 to +0.0035 delta-AUROC after

explicit covariate adjustment (3/6 domain-splits Fisher-

significant); H70 triangle-defect is 93% coexpression-independent

on a fresh OLS partial-out test (immune middle layer); H123

motif-community is best understood as a composite, and the

manuscript's H124-H138 cascade documents how adding coexpression-

correlated annotations monotonically erodes robustness. Full table

and fresh test: revision_experiments/r5_coexpression_residual/.

--------------------------------------------------------------------------------

Section 3.4 / 4.4 -- Ethical, practical, scalability considerations

--------------------------------------------------------------------------------

REVIEWER COMMENT

"Ethical considerations... biases in biological datasets...

computational cost and scalability of the hypothesis-screening

framework... integration into biological pipelines or clinical

applications."

RESPONSE

The Discussion now contains three new short subsections:

"Computational footprint and scalability" reports the per-iteration

wall-clock (mean 14.7 min executor; 95th percentile 19.7 min), total

campaign cost (~16 h on a single workstation plus high-effort LLM

agent), and an asymptotic cost analysis (O(N * G^3) in null replicates

and gene count for the persistent-homology component). Full timings

are parsed from iterations/iter_*/iteration_meta.json and saved to

revision_experiments/r6_scalability/.

"Pathways to practical use" describes two near-term applications:

geometric-prior reweighting of GRNBoost-style network inference, and

regulator prioritisation via cross-model conserved community

membership.

"Ethical considerations" addresses the demographic and gene-coverage

biases inherited from Tabula Sapiens-style atlases and the DoRothEA /

TRRUST / STRING / GO databases, and cautions against clinical

extrapolation. We also note the dual-use risk of autonomous-screening

frameworks generally and frame the comprehensive reporting of all 141

hypotheses (including 70+ negatives) as a deliberate guard.

================================================================================

SUMMARY OF NEW CONTENT

================================================================================

The revision adds approximately 1.5 pages of body text, six new

supplementary analysis directories, and one supplementary table; no

existing figures or numerical claims were removed. All quantitative

revisions are reproducible from the scripts under

revision_experiments/r1_kidney_replication/

revision_experiments/r2_multiple_comparisons/

revision_experiments/r3_h123_ablation/

revision_experiments/r4_cv_hparam/

revision_experiments/r5_coexpression_residual/

revision_experiments/r6_scalability/

Attachment Submitted filename: Response_to_Reviewers.pdf

# Editor report: Decision Letter 1

**DOI:** 10.1371/journal.pone.0344826.r003

**Authors:** Alexander Kolpakov

30 Jun 2026

<p>What topological and geometric structure do biological foundation models learn? Evidence from 141 hypotheses

PONE-D-26-09692R1

Dear Dr. Kendiukhov,

We’re pleased to inform you that your manuscript has been judged scientifically suitable for publication and will be formally accepted for publication once it meets all outstanding technical requirements.

Within one week, you’ll receive an e-mail detailing the required amendments. When these have been addressed, you’ll receive a formal acceptance letter and your manuscript will be scheduled for publication.

An invoice will be generated when your article is formally accepted. Please note, if your institution has a publishing partnership with PLOS and your article meets the relevant criteria, all or part of your publication costs will be covered. Please make sure your user information is up-to-date by logging into Editorial Manager at Editorial Manager® and clicking the ‘Update My Information' link at the top of the page. For questions related to billing, please contact billing support.

If your institution or institutions have a press office, please notify them about your upcoming paper to help maximize its impact. If they’ll be preparing press materials, please inform our press team as soon as possible -- no later than 48 hours after receiving the formal acceptance. Your manuscript will remain under strict press embargo until 2 pm Eastern Time on the date of publication. For more information, please contact onepress@plos.org.

Kind regards,

Alexander Kolpakov

Academic Editor

PLOS One

Additional Editor Comments (optional):

Reviewers' comments:

# Editor report: Acceptance letter

**DOI:** 10.1371/journal.pone.0344826.r004

**Authors:** Alexander Kolpakov

PONE-D-26-09692R1

PLOS One

Dear Dr. Kendiukhov,

I'm pleased to inform you that your manuscript has been deemed suitable for publication in PLOS One. Congratulations! Your manuscript is now being handed over to our production team.

At this stage, our production department will prepare your paper for publication. This includes ensuring the following:

* All references, tables, and figures are properly cited

* All relevant supporting information is included in the manuscript submission,

* There are no issues that prevent the paper from being properly typeset

You will receive further instructions from the production team, including instructions on how to review your proof when it is ready. Please keep in mind that we are working through a large volume of accepted articles, so please give us a few days to review your paper and let you know the next and final steps.

Lastly, if your institution or institutions have a press office, please let them know about your upcoming paper now to help maximize its impact. If they'll be preparing press materials, please inform our press team within the next 48 hours. Your manuscript will remain under strict press embargo until 2 pm Eastern Time on the date of publication. For more information, please contact onepress@plos.org.

You will receive an invoice from PLOS for your publication fee after your manuscript has reached the completed accept phase. If you receive an email requesting payment before acceptance or for any other service, this may be a phishing scheme. Learn how to identify phishing emails and protect your accounts at https://explore.plos.org/phishing.

If we can help with anything else, please email us at customercare@plos.org.

Thank you for submitting your work to PLOS ONE and supporting open access.

Kind regards,

PLOS ONE Editorial Office Staff

on behalf of

Dr. Alexander Kolpakov

Academic Editor

PLOS One
